# Evaluation of Alpha-Synuclein Cerebrospinal Fluid Levels in Several Neurological Disorders

**DOI:** 10.3390/jcm11113139

**Published:** 2022-05-31

**Authors:** Luisa Agnello, Bruna Lo Sasso, Matteo Vidali, Concetta Scazzone, Caterina Maria Gambino, Tommaso Piccoli, Giulia Bivona, Anna Maria Ciaccio, Rosaria Vincenza Giglio, Vincenzo La Bella, Marcello Ciaccio

**Affiliations:** 1 Institute of Clinical Biochemistry, Clinical Molecular Medicine and Clinical Laboratory Medicine, Department of Biomedicine, Neurosciences and Advanced Diagnostics, University of Palermo, 90127 Palermo, Italy; luisa.agnello@unipa.it (L.A.); bruna.losasso@unipa.it (B.L.S.); concetta.scazzone@unipa.it (C.S.); caterinamaria.gambino@unipa.it (C.M.G.); giulia.bivona@unipa.it (G.B.); rosariavincenza.giglio@unipa.it (R.V.G.); 2 Department of Laboratory Medicine, Azienda Ospedaliera Universitaria Policlinico “P. Giaccone”, 90127 Palermo, Italy; 3 Foundation IRCCS Ca’ Granda Ospedale Maggiore Policlinico, 20122 Milan, Italy; matteo.vidali@gmail.com; 4 Unit of Neurology, Department of Biomedicine, Neuroscience and Advanced Diagnostics, University of Palermo, 90127 Palermo, Italy; tommaso.piccoli@unipa.it; 5 Unit of Clinical Biochemistry, University of Palermo, 90127 Palermo, Italy; annamaria.ciaccio@unipa.it; 6 ALS Clinical Research Center and Laboratory of Neurochemistry, Department of Biomedicine, Neuroscience and Advanced Diagnostics, University of Palermo, 90127 Palermo, Italy; vincenzo.labella@unipa.it

**Keywords:** CSF, biomarker, parkinson’s disease, alzheimer’s disease, neurodegeneration

## Abstract

(1) Background: Alpha-synuclein (α-syn) is a presynaptic neuronal protein that regulates several neuronal functions. In recent decades, the role of α-syn as a biomarker of neurodegenerative diseases has been explored, especially in synucleinopathies. However, only a few studies have assessed its role as biomarker in other neurological disorders. The aim of the study was to evaluate cerebrospinal fluid (CSF) α-syn levels in several neurological disorders; (2) Methods: We measured CSF α-syn levels by a commercial ELISA kit in 158 patients classified in the following group: controls, Alzheimer’s Disease (AD), cerebrovascular diseases, inflammatory central nervous system diseases, other neurological diseases, Parkinson’s Disease (PD), and peripheral neuropathy; (3) Results: Patients with PD showed the lowest and patients with AD the highest levels of CSF α-syn (1372 vs. 2912 pg/mL, respectively, *p* < 0.001). In AD patients, α-syn levels were significantly associated with tau proteins; (4) Conclusions: α-syn could represent a biomarker of neurodegenerative diseases.

## 1. Introduction

Alpha-synuclein (α-syn) is a protein belonging to the synuclein family, consisting of three small soluble proteins, namely alpha-, beta-, and gamma- [1]. Although α-syn is present in different cell types, including erythrocytes and platelets, it is predominantly expressed in the cytoplasm of neuronal cells [2]. Under physiological conditions, it is ubiquitously expressed in the pre-synaptic terminal, where it interacts with membranes of synaptic vesicles, contributing to neurotransmission and synaptic homeostasis [3].

α-syn physiologically exists in a dynamic equilibrium between cytosolic monomeric unfolded forms and helically folded tetramers bound to membranes. Under pathological conditions, α-syn monomers could aggregate into insoluble fibrils, known as Lewy Bodies (LBs) and Lewy Neurites (LNs), which represent the pathological hallmark of synucleinopathies, such as Parkinson’s Disease (PD), dementia with LBs (DLBs), and multiple system atrophy (MSA) [4]. Although α-syn is abundant in neuronal cytoplasm, its presence in the extracellular space has also been detected under both physiological and pathological conditions [5,6,7]. El-Agnaf et al. detected α-syn in human CSF first [8]. Since then, CSF α-Syn levels have been evaluated in several studies, and some authors found decreased CSF α-syn levels in PD patients in comparison to healthy controls and other neurodegenerative disorders [9,10,11,12]. Studies on other synucleinopathies, such as DLBs and MSA, achieved contrasting results [7,13,14].

On the other hand, increased levels of CSF α-syn have been detected in Alzheimer’s Disease (AD) patients, suggesting that it could represent a biomarker of neurodegeneration in a similar way to high levels of CSF tau [15]. However, other authors found no alteration or decreased levels of α-syn in AD patients [16,17]. Thus, a definite conclusion on the role of α-syn in AD cannot be drawn. 

Beyond the role of α-syn in PD and AD, increased literature evidence suggests that it could be involved in the pathogenesis of several other neurological disorders [18]. It has been hypothesized that α-syn could be a potentiator of neurodegeneration. Indeed, it is abundantly expressed in synapses and could be involved in synaptic dysfunction, which represents a common feature of neurodegenerative diseases [19]. 

The aim of this retrospective observational study was to assess the CSF total α-syn levels in a large population of patients with different neurological disorders. 

## 2. Materials and Methods

### 2.1. Study Population

In this study performed at the Palermo University Hospital “P Giaccone”, we enrolled patients with different neurological disorders attending the Unit of Neurology from 2000 to 2020, who underwent lumbar puncture for CSF analysis as part of their diagnostic evaluation. The diagnosis was made by an experienced neurologist based on clinical and laboratory findings. 

All the clinical and biological assessments were carried out in accordance with the Declaration of Helsinki and its amendments.

### 2.2. CSF Analysis

The CSF samples were collected between 8:00 a.m. and 10:00 a.m. from fasted patients and were labeled to ensure anonymity. Specifically, the CSF was obtained by a lumbar puncture at the L3/4 or L4/5 interspace using a 21-gauge needle, collected in polypropylene tubes, centrifuged at 500 g for 20 min, aliquoted in propylene tubes and stored at −80 °C until analysis, according to international consensus protocols [20].

CSF α-syn levels were measured by a commercial ELISA kit (Euroimmun, Lübeck, Germany), according to manufacturer’s instructions.

The CSF beta amyloid 42 (β42), β40, phosphorylated tau at threonine 181 (P-tau) and total tau (T-tau) levels were measured by chemiluminescence enzyme immunoassay (CLEIA) on a fully automated platform (Lumipulse G1200 analyzer, Fujirebio Inc. Europe, Gent, Belgium) according to the manufacturer.

### 2.3. Statistical Analysis

Statistical analyses were performed by SPSS statistical software v.17.0 (SPSS Inc., Chica-go, IL, USA) and R Language v.4.0.3 (R Foundation for Statistical Computing, VI, Austria). Normality distribution was assessed preliminarily by q-q plot and Shapiro–Wilk tests. Quantitative variables were expressed by the median and interquartile range (IQR), while qualitative variables as absolute and relative frequencies. Differences among groups for continuous variables were estimated by nonparametric Kruskal–Wallis test (if >2 groups) or Mann–Whitney U-test (with Holm-Bonferroni’s correction for multiple comparisons). The association between quantitative variables was evaluated by nonparametric Spearman’s rank-order correlation.

## 3. Results

One hundred and fifty-eight subjects were included. They were sub-grouped as controls (n = 35), including psychiatric disorders, AD (n = 25), cerebrovascular diseases (n = 18), inflammatory central nervous system (CNS) diseases (n = 10), other neurological diseases (n = 7), including epilepsy and brain cancer, PD (n = 22), and peripheral neuropathy (n = 41).

**Figure 1:**
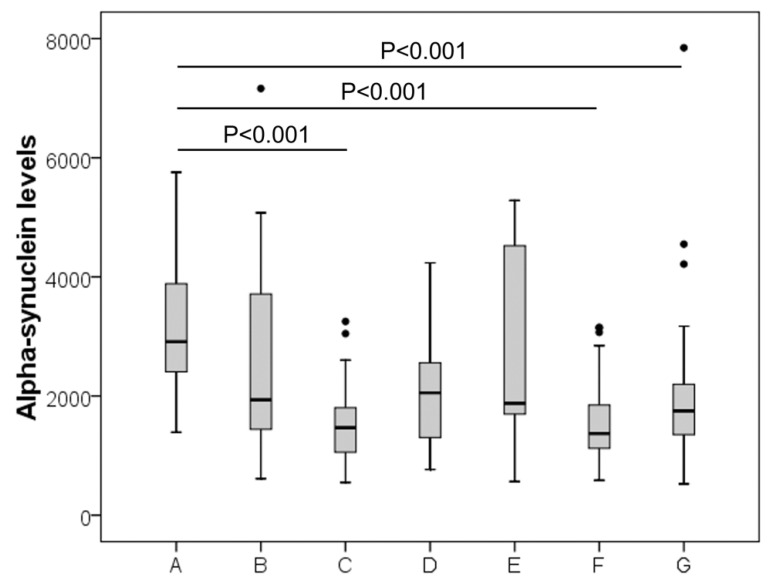
Distribution of CSF α-Synuclein levels in the study population. A (Alzheimer), B (cerebrovascular disease), C (controls), D (inflammatory CNS disease), E (other neurological diseases), F (Parkinson’s Disease) and G (peripheral neuropathy).

Demographic characteristics (sex and age) are shown in Table 1. α-Syn levels were evaluated and shown to be significantly different among groups (overall Kruskal–Wallis test *p* < 0.001) (Figure 1). In particular, considering the Holm-Bonferroni’s correction for multiple comparisons, patients with AD displayed significantly higher median α-Syn levels than controls (2912 vs. 1469 pg/mL; *p* < 0.001), PD (2912 vs. 1372 pg/mL; *p* < 0.001) and peripheral neuropathy (2912 vs. 1751 pg/mL; *p* < 0.001) (Figure 1). Among the other 18 post hoc comparisons, only other 4 *p*-values were lower than 0.05 (AD vs. Inflammatory CNS diseases *p* = 0.017; AD vs. Cerebrovascular diseases *p* = 0.022; Controls vs. Peripheral Neuropathy *p* = 0.039 and Controls vs. Other neurological diseases *p* = 0.049) but they did not pass the Holm-Bonferroni’s correction criteria.

CSF levels of AD core biomarkers, including (T-tau), (P-tau), β42, β40 and β42/40 ratio, were further evaluated in the AD. α-syn levels were found to be associated at varying extents to T-tau (rho = 0.630, *p* = 0.002), P-tau (rho = 0.498, *p* = 0.018), β42 (rho = 0.485, *p* = 0.022), β40 (rho = 0.488, *p* = 0.021). No association was instead observed between α-syn and β42/40 ratio (rho = 0.222, *p* = 0.320). Other statistically significant associations were found between T-tau and P-tau (rho = 0.785, *p* < 0.001), P-tau and β40 (rho = 0.450, *p* = 0.036), β42 and β40 (rho = 0.755, *p* < 0.001), β42 and β42/40 ratio (rho = 0.622, *p* = 0.002) (Table 2).

## 4. Discussion

Neurological disorders represent an important health burden, especially considering the progressive increase in aging in the general population. Their early identification is crucial, but still today, it is challenging. CSF biomarkers represent precious tools for assessing neurodegenerative disorders providing in vivo information on the underlying pathology [21,22,23,24]. Specifically, CSF is an ideal biofluid due to its proximity to the brain parenchyma, the moderately low cost in comparison to positron emission tomography imaging, and the safety of lumbar puncture. To date, CSF biomarkers have been implemented in the diagnostic work-up for AD [25,26]. Specifically, the detection of decreased levels of β42 and β42/40 ratio, and increased levels of T-tau and P-tau in the CSF support the diagnosis of AD [27,28]. Over the past ten years, research on biomarkers in synucleinopathies has markedly expanded. Among these, CSF α-syn has gained significant attention, but more evidence is still required before it is used in clinical practice.

In this study, we sought to evaluate the total CSF α-syn levels in a large population of individuals with different neurological disorders. The main findings of our study can be summarized as follows: (i) CSF α-syn levels were significantly different across all groups. Specifically, AD patients showed the highest levels, while PD patients the lowest; (ii) in AD patients, α-syn levels were significantly associated with T-tau, P-tau, β42 and β40. Overall, our findings suggest that α-syn could represent a biomarker of neurodegenerative diseases. Specifically, CSF α-syn could be used in clinical practice as a specific biomarker of PD, which is characterized by decreased α-syn levels, and as an unspecific biomarker of synaptic degeneration, which is characterized by increased levels. However, more studies are required for unraveling the molecular underpinnings of the alteration of CSF α-syn levels in neurodegenerative diseases.

To date, several authors have assessed the potential role of α-syn as a biomarker of PD [29]. Findings from four meta-analyses agree that PD patients have decreased levels of α-syn in comparison to controls [9,30,31,32]. α-syn could support the differential diagnosis between PD and other neurodegenerative disorders, but it is not associated with the PD severity [33]. However, some limitations hamper its clinical use. High heterogeneity in CSF α-syn levels among studies has been reported [10]. Such a discrepancy could be due to the different characteristics of PD patients enrolled in each study, as well as the control population, which vary from healthy controls to patients with other neurological diseases.

Fewer studies evaluated the role of α-syn in AD patients, achieving inconsistent results. Our results are in accordance with some authors who found increased levels of α-syn in AD patients than controls and patients with other neurological diseases [34,35,36]. It has been hypothesized that α-syn interacts with amyloid and tau, enhancing neurodegeneration. In accordance with such a hypothesis, in our study we found that α-syn levels were significantly associated with T-tau and P-tau. Remarkably, α-syn and tau share an important role in cell trafficking and synaptic functions, as well as in controlling mitochondrial homeostasis [37]. Thus, the alterations in α-syn and tau levels are indicative of synapsis loss and disruption.

Mutual interaction of α-syn with β-amyloid and tau, which promotes neurodegeneration and worse prognosis, has been hypothesized in AD patients [38]. Thus, it has been suggested that CSF α-syn could improve the prognostic performance of the AD biomarker panel. Larson et al. showed that α-syn has a stronger correlation with the degree of cognitive impairment than tau [39]. Additionally, the co-expression of α-syn and β amyloid is associated with a more aggressive cognitive decline in AD patients [40]. Thus, evaluating the three proteins, namely β amyloid, tau, and α-syn could provide complementary diagnostic and prognostic information. This could have implications for the development of disease-modifying therapies.

To the best of our knowledge, we first evaluated α-syn in a wide range of neurological diseases, revealing that, except in PD, its levels are increased in all these conditions in comparison with controls. This finding supports the hypothesis that α-syn could play a role in the pathogenesis of neurological disorders [18]. However, further studies are required to confirm such a preliminary finding.

Overall, CSF α-syn levels could depend on competing processes. On one side, decreased levels could be the result of its intracellular sequestration into LBs or LNs, therefore reducing the amount of protein available for the physiological release into the extracellular compartment, as observed in patients with PD. On the other side, the release of α-syn from degenerating synapses could lead to an increase in its CSF levels, as observed in AD [41].

The limitation and strengths of our study must be mentioned. The small number of patients per study group is the main limitation, while the well-characterized patient groups, the use of a validated assay and the appropriate pre-analytical sample handling represent the strengths of our study.

In conclusion, α-syn could represent a diagnostic biomarker of PD because its levels are typically decreased, while it could have a prognostic value in other neurological disorders, including AD, which is characterized by an increase in CSF α-syn levels [42]. Although α-syn is a promising biomarker of neurological disorders, further efforts are required before introducing it in clinical practice, such as the harmonization and standardization of the assays for its measurement. Indeed, high heterogeneity among studies exists and could be attributed to several factors, including peri-analytical and analytical aspects, such as CSF collection and storage, as well as the type of capture and detection antibodies used [11].

## Figures and Tables

**Table 1:** Demographic characteristics of study population and α-synuclein levels.

Group	Sex, M% (Total nr)	Age (Median, IQR)	α-syn, pg/mL (Median, IQR)
AD	56 (25)	73 (67–76)	2912 (2243–4036)
Cerebrovascular diseases	67 (18)	60 (53–70)	1938 (1337–3719)
Controls	54 (35)	51 (37–66)	1469 (1047–1827)
Inflammatory CNS diseases	30 (10)	64 (58–76)	2053 (1284–2677)
Other neurological diseases	43 (7)	63 (48–72)	1879 (1679–4698)
PD	64 (22)	65 (56–72)	1372 (1103–2031)
Peripheral Neuropathy	61 (41)	60 (48–71)	1751 (1333–2225)

AD, Alzheimer’s Disease; CNS, central nervous system; PD, Parkinson’s disease; IQR, interquartile range.

**Table 2:** Correlation analysis between biomarkers.

	α-syn	T-tau	P-tau	β42	β40	β42/40 Ratio
**α-synuclein**		0.630 ***p* = 0.002**	0.498 ***p* = 0.018**	0.485 ***p* = 0.022**	0.488 ***p* = 0.021**	0.222 *p* = 0.320
**T-tau**			0.785 ***p* < 0.001**	0.291 *p* = 0.189	0.309 *p* = 0.162	0.040 *p* = 0.859
**P-tau**				0.233 *p* = 0.296	0.450 ***p* = 0.036**	−0.142 *p* = 0.527
**β42**					0.755 ***p* < 0.001**	0.622 ***p* = 0.002**
**β40**						0.028 *p* = 0.903
**β42/40 ratio**						

## Data Availability

The datasets generated during and/or analysed during the current study are available from the corresponding author on reasonable request.

## References

[1] Clayton D.F., George J.M. (1998). The synucleins: A family of proteins involved in synaptic function, plasticity, neurodegeneration and disease. Trends Neurosci..

[2] Del Tredici K., Braak H. (2016). Review: Sporadic Parkinson’s disease: Development and distribution of α-synuclein pathology. Neuropathol. Appl. Neurobiol..

[3] Burré J. (2015). The Synaptic Function of α-Synuclein. J. Park. Dis..

[4] Mahul-Mellier A.L., Burtscher J., Maharjan N., Weerens L., Croisier M., Kuttler F., Leleu M., Knott G.W., Lashuel H.A. (2020). The process of Lewy body formation, rather than simply α-synuclein fibrillization, is one of the major drivers of neurodegeneration. Proc. Natl. Acad. Sci. USA.

[5] Paillusson S., Clairembault T., Biraud M., Neunlist M., Derkinderen P. (2013). Activity-dependent secretion of alpha-synuclein by enteric neurons. J. Neurochem..

[6] Emmanouilidou E., Elenis D., Papasilekas T., Stranjalis G., Gerozissis K., Ioannou P.C., Vekrellis K. (2011). Assessment of α-synuclein secretion in mouse and human brain parenchyma. PLoS ONE.

[7] Mollenhauer B., Locascio J.J., Schulz-Schaeffer W., Sixel-Döring F., Trenkwalder C., Schlossmacher M.G. (2011). α-Synuclein and tau concentrations in cerebrospinal fluid of patients presenting with parkinsonism: A cohort study. Lancet Neurol..

[8] El-Agnaf O.M., Salem S.A., Paleologou K.E., Cooper L.J., Fullwood N.J., Gibson M.J., Curran M.D., Court J.A., Mann D.M., Ikeda S. (2003). Alpha-synuclein implicated in Parkinson’s disease is present in extracellular biological fluids, including human plasma. FASEB J..

[9] Eusebi P., Giannandrea D., Biscetti L., Abraha I., Chiasserini D., Orso M., Calabresi P., Parnetti L. (2017). Diagnostic utility of cerebrospinal fluid α-synuclein in Parkinson’s disease: A systematic review and meta-analysis. Mov. Disord..

[10] Parnetti L., Gaetani L., Eusebi P., Paciotti S., Hansson O., El-Agnaf O., Mollenhauer B., Blennow K., Calabresi P. (2019). CSF and blood biomarkers for Parkinson’s disease. Lancet Neurol..

[11] Constantinides V.C., Majbour N.K., Paraskevas G.P., Abdi I., Safieh-Garabedian B., Stefanis L., El-Agnaf O.M., Kapaki E. (2021). Cerebrospinal Fluid α-Synuclein Species in Cognitive and Movements Disorders. Brain Sci..

[12] Agnello L., Gambino C.M., Lo Sasso B., Bivona G., Milano S., Ciaccio A.M., Piccoli T., La Bella V., Ciaccio M. (2021). Neurogranin as a Novel Biomarker in Alzheimer’s Disease. Lab. Med..

[13] Shi M., Bradner J., Hancock A.M., Chung K.A., Quinn J.F., Peskind E.R., Galasko D., Jankovic J., Zabetian C.P., Kim H.M. (2011). Cerebrospinal fluid biomarkers for Parkinson disease diagnosis and progression. Ann. Neurol..

[14] Wang Y., Shi M., Chung K.A., Zabetian C.P., Leverenz J.B., Berg D., Srulijes K., Trojanowski J.Q., Lee V.M., Siderowf A.D. (2012). Phosphorylated α-synuclein in Parkinson’s disease. Sci. Transl. Med..

[15] Compta Y., Revesz T. (2021). Neuropathological and Biomarker Findings in Parkinson’s Disease and Alzheimer’s Disease: From Protein Aggregates to Synaptic Dysfunction. J. Park. Dis..

[16] Lee Y.G., Jeon S., Kang S.W., Park M., Baik K., Yoo H.S., Chung S.J., Jeong S.H., Jung J.H., Lee P.H. (2021). Interaction of CSF α-synuclein and amyloid beta in cognition and cortical atrophy. Alzheimer’s Dement. Diagn. Assess. Dis. Monit..

[17] Mackin R.S., Insel P., Zhang J., Mohlenhoff B., Galasko D., Weiner M., Mattsson N. (2015). Cerebrospinal fluid α-synuclein and Lewy body-like symptoms in normal controls, mild cognitive impairment, and Alzheimer’s disease. J. Alzheimers Dis..

[18] Visanji N.P., Lang A.E., Kovacs G.G. (2019). Beyond the synucleinopathies: Alpha synuclein as a driving force in neurodegenerative comorbidities. Transl. Neurodegener..

[19] Bae J.R., Kim S.H. (2017). Synapses in neurodegenerative diseases. BMB Rep..

[20] del Campo M., Mollenhauer B., Bertolotto A., Engelborghs S., Hampel H., Simonsen A.H., Kapaki E., Kruse N., Le Bastard N., Lehmann S. (2012). Recommendations to standardize preanalytical confounding factors in Alzheimer’s and Parkinson’s disease cerebrospinal fluid biomarkers: An update. Biomark. Med..

[21] Allegri R.F. (2020). Moving from neurodegenerative dementias, to cognitive proteinopathies, replacing “where” by “what”…. Dement. Neuropsychol..

[22] Camporesi E., Nilsson J., Brinkmalm A., Becker B., Ashton N.J., Blennow K., Zetterberg H. (2020). Fluid Biomarkers for Synaptic Dysfunction and Loss. Biomark. Insights.

[23] Agnello L., Colletti T., Lo Sasso B., Vidali M., Spataro R., Gambino C.M., Giglio R.V., Piccoli T., Bivona G., La Bella V. (2021). Tau protein as a diagnostic and prognostic biomarker in amyotrophic lateral sclerosis. Eur. J. Neurol..

[24] Colletti T., Agnello L., Spataro R., Guccione L., Notaro A., Lo Sasso B., Blandino V., Graziano F., Gambino C.M., Giglio R.V. (2021). Prognostic Role of CSF β-amyloid 1-42/1-40 Ratio in Patients Affected by Amyotrophic Lateral Sclerosis. Brain Sci..

[25] Dubois B., Feldman H.H., Jacova C., Hampel H., Molinuevo J.L., Blennow K., DeKosky S.T., Gauthier S., Selkoe D., Bateman R. (2014). Advancing research diagnostic criteria for Alzheimer’s disease: The IWG-2 criteria. Lancet Neurol..

[26] McKhann G.M., Knopman D.S., Chertkow H., Hyman B.T., Jack C.R., Kawas C.H., Klunk W.E., Koroshetz W.J., Manly J.J., Mayeux R. (2011). The diagnosis of dementia due to Alzheimer’s disease: Recommendations from the National Institute on Aging-Alzheimer’s Association workgroups on diagnostic guidelines for Alzheimer’s disease. Alzheimers Dement..

[27] Agnello L., Piccoli T., Vidali M., Cuffaro L., Lo Sasso B., Iacolino G., Giglio V.R., Lupo F., Alongi P., Bivona G. (2020). Diagnostic accuracy of cerebrospinal fluid biomarkers measured by chemiluminescent enzyme immunoassay for Alzheimer disease diagnosis. Scand. J. Clin. Lab. Investig..

[28] McGrowder D.A., Miller F., Vaz K., Nwokocha C., Wilson-Clarke C., Anderson-Cross M., Brown J., Anderson-Jackson L., Williams L., Latore L. (2021). Cerebrospinal Fluid Biomarkers of Alzheimer’s Disease: Current Evidence and Future Perspectives. Brain Sci..

[29] Twohig D., Nielsen H.M. (2019). α-synuclein in the pathophysiology of Alzheimer’s disease. Mol. Neurodegener..

[30] Sako W., Murakami N., Izumi Y., Kaji R. (2014). Reduced alpha-synuclein in cerebrospinal fluid in synucleinopathies: Evidence from a meta-analysis. Mov. Disord..

[31] Zhou B., Wen M., Yu W.F., Zhang C.L., Jiao L. (2015). The Diagnostic and Differential Diagnosis Utility of Cerebrospinal Fluid α -Synuclein Levels in Parkinson’s Disease: A Meta-Analysis. Park. Dis..

[32] Gao L., Tang H., Nie K., Wang L., Zhao J., Gan R., Huang J., Zhu R., Feng S., Duan Z. (2015). Cerebrospinal fluid alpha-synuclein as a biomarker for Parkinson’s disease diagnosis: A systematic review and meta-analysis. Int. J. Neurosci..

[33] Chalatsa I., Melachroinou K., Emmanouilidou K.E., Vekrellis E.K. (2020). Assessment of cerebrospinal fluid α-synuclein as a potential biomarker in Parkinson’s disease and synucleinopathies. Neuroimmunol. Neuroinflamm..

[34] Wang Z.Y., Han Z.M., Liu Q.F., Tang W., Ye K., Yao Y.Y. (2015). Use of CSF α-synuclein in the differential diagnosis between Alzheimer’s disease and other neurodegenerative disorders. Int. Psychogeriatr..

[35] Chiasserini D., Biscetti L., Eusebi P., Salvadori N., Frattini G., Simoni S., De Roeck N., Tambasco N., Stoops E., Vanderstichele H. (2017). Differential role of CSF fatty acid binding protein 3, α-synuclein, and Alzheimer’s disease core biomarkers in Lewy body disorders and Alzheimer’s dementia. Alzheimers Res. Ther..

[36] Majbour N.K., Chiasserini D., Vaikath N.N., Eusebi P., Tokuda T., van de Berg W., Parnetti L., Calabresi P., El-Agnaf O.M. (2017). Increased levels of CSF total but not oligomeric or phosphorylated forms of alpha-synuclein in patients diagnosed with probable Alzheimer’s disease. Sci. Rep..

[37] Vacchi E., Kaelin-Lang A., Melli G. (2020). Tau and Alpha Synuclein Synergistic Effect in Neurodegenerative Diseases: When the Periphery Is the Core. Int. J. Mol. Sci..

[38] Jellinger K.A. (2011). Interaction between α-synuclein and other proteins in neurodegenerative disorders. Sci. World J..

[39] Larson M.E., Sherman M.A., Greimel S., Kuskowski M., Schneider J.A., Bennett D.A., Lesné S.E. (2012). Soluble α-synuclein is a novel modulator of Alzheimer’s disease pathophysiology. J. Neurosci..

[40] Clinton L.K., Blurton-Jones M., Myczek K., Trojanowski J.Q., LaFerla F.M. (2010). Synergistic Interactions between Abeta, tau, and alpha-synuclein: Acceleration of neuropathology and cognitive decline. J. Neurosci..

[41] Mollenhauer B., Bowman F.D., Drake D., Duong J., Blennow K., El-Agnaf O., Shaw L.M., Masucci J., Taylor P., Umek R.M. (2019). Antibody-based methods for the measurement of α-synuclein concentration in human cerebrospinal fluid-method comparison and round robin study. J. Neurochem..

[42] Rong H., Jin L., Wei W., Wang X., Xi Z. (2015). Alpha-synuclein is a potential biomarker in the serum and CSF of patients with intractable epilepsy. Seizure.

